# An Account of the Good Effects of Electricity in Four Cases of a Diseased Testicle

**Published:** 1786

**Authors:** George Hounsfield

**Affiliations:** Surgeon at Sheffield in Yorkshire, and Member of the Corporation of Surgeons of London


### II. *An Account of the good Effects of Electricity in four Cases of a diseased Testicle. Communicated in a Letter to Dr.* Simmons *by Mr.* George Hounsfield, *Surgeon at Sheffield in Yorkshire, and Member of the Corporation of Surgeons of London.*

MR. Hunter, in his late treatife on the Venereal Difeafe, having made mention of the advantages which may be derived from the ufe of electricity in fome affections of the teftis, the following cafes of its fingular fuccefs may perhaps appear to you fufficiently interefting to merit a place in the London Medical Journal.

### CASE I.

A dragoon was admitted into St. Thomas's Hofpital, in London, under the care of Mr. Birch, with a confiderable enlargement of one of his tefticles, which had proceeded from a blow againft the pummel of a faddle fome months before. The body of the teftis was very hard, and much enlarged; but the fpermatic chord was of its natural fize, and free from difeafe. Mercurial ointment was directed to be rubbed on the part; cataplafms were likewife applied, and emetics repeatedly adminiftered,

ſtered, but without producing any alteration in the complaint. At length, caſtration was propoſed; but previouſly to the having recourſe to this operation, I was directed to paſs electric ſhocks through the part. In a few days a ſenſible alteration for the better was perceived, and in a fortnight the teſtis could be diſtinguiſhed by the finger from the epidydimis, and was much ſofter. The ſhocks were daily continued for ſix weeks; at the end of which period the patient was diſcharged, perfectly cured.

### CASE II.

Thomas Pitt was admitted into St. Thomas's Hoſpital, under Mr. Birch's care, with a hardneſs of one of the teſticles, much more conſiderable than that mentioned in the preceding caſe. The complaint in this patient ſeemed to have been occaſioned by the neglect of a venereal affection. I was directed to paſs a very ſtrong electric ſhock through it; and this brought on conſiderable heat in the part. Similar ſhocks were repeated at the intervals of three days for four times.

Under this treatment, the teſtis grew ſofter; at length a fluctuation was perceived, and an abſceſs abſceſs burſt externally, and afterwards cloſed. Electric ſhocks of a more moderate ſtrength were now paſſed through the part; a ſecond abſceſs formed, and each time, after the ſhocks were adminiſtered, it diſcharged more than on the days they were not paſſed. The teſtis was, by degrees, ſo much reduced in ſize and hardneſs, that the patient was permitted to go to work, on condition of his continuing to apply to me every Sunday, when I gave him a few ſhocks. This caſe was tedious; but, at the end of ſix months, the hardneſs was completely diſſolved, and the abſceſs healed.

### CASE III.

A ſerjeant of the Suſſex militia was admitted into the ſame hoſpital, with a ſchirrous teſtis; the extirpation of which had been adviſed. In this caſe, after the uſe of emetics, and the local application of mercury and cataplaſms had been tried without effect, the ſame mode of treatment was adopted as in the preceding caſes; and the patient was perfectly cured.

When I paſſed a ſtrong ſhock through the teſtis, in this caſe, the patient was ſo much affected by it as to be feveriſh and indiſpoſed, and I was obliged to omit the uſe of electricity for ſome days, and to be more gentle in my re-application of it: the teſtis, however, was ſofter. I afterwards repeated a ſtrong ſhock, to try if it would be followed with the ſame conſequences as before. The effects were the ſame; and I therefore adhered to the uſe of more moderate ſhocks; by which means the cure was accompliſhed in about ten weeks.

### CASE IV.

A black ſervant was admitted into the ſame hoſpital, with an enlarged teſtis; but with the epidydimis more indurated than the teſtis. This was treated, by direction of Mr. Birch, with electric ſhocks, and was cured in two months.

In this, as well as in all the other caſes, the teſticle would, in the end, have been extirpated, if the powers of electricity had not proved efficacious.

*Sheffield, Yorkſhire,*

June 27, 1786.

III. *Caſe*

